# Prescription patterns of traditional Chinese medicine amongst Taiwanese children: a population-based cohort study

**DOI:** 10.1186/s12906-018-2261-2

**Published:** 2018-06-22

**Authors:** Hwey-Fang Liang, Yao-Hsu Yang, Pau-Chung Chen, Hsing-Chun Kuo, Chia-Hao Chang, Ying-Hsiang Wang, Kuang-Ming Wu

**Affiliations:** 1 Department of Nursing, Chang Gung University of Science and Technology, No.2, Sec. W., Jiapu Rd, Puzi City, Chiayi County 61363 Taiwan; 2 Chronic Diseases and Health Promotion Research Center, Chang Gung University of Science and Technology, No.2, Sec. W., Jiapu Rd, Puzi City, Chiayi County 61363 Taiwan; 3 Chang Gung Memorial Hospital, Chiayi, No.6, Sec. W., Jiapu Rd, Puzi City, Chiayi County 61363 Taiwan; 4 Department for Traditional Chinese Medicine, Chang Gung Memorial Hospital, Chiayi, Taiwan; 5 Health Information and Epidemiology Laboratory of Chang Gung Memorial Hospital, Chiayi, Taiwan; 6 School of Traditional Chinese Medicine, College of Medicine, Chang Gung University, Taoyuan, Taiwan; 7 Institute of Occupational Medicine and Industrial Hygiene, National Taiwan University College of Public Health, Taipei, Taiwan; 8 Department of Environmental and Occupational Medicine, National Taiwan University College of Medicine and National Taiwan University Hospital, Taipei, Taiwan; 9 Department of Pediatrics, Chang Gung Memorial Hospital, Chiayi, Taiwan; 10 Department of Early Childhood Education, National Chiayi University, Chiayi, Taiwan

**Keywords:** Traditional Chinese medicine, Chinese herbal remedies, Herbal formulae, Single herb

## Abstract

**Background:**

Traditional Chinese medicine (TCM) has been used by Chinese patients and in many other countries worldwide. However, epidemiological reports and prescription patterns on children are few.

**Methods:**

A cohort of 178,617 children aged 18 and under from one million randomly sampled cases of the National Health Insurance Research Database was analyzed for TCM prescription patterns. SAS 9.1 was applied and descriptive medicine utilization patterns were presented.

**Results:**

The cohort included 112,889 children treated by TCM, with adolescents (12- to 18-year-olds) as the largest group. In the children’s TCM outpatient visits, Chinese herbal remedies were the main treatment. The top three categories of diseases treated with Chinese herbal remedies were respiratory system; symptoms, signs, and ill-defined conditions; and digestive system. The top three categories using acupuncture were: injury and poisoning, diseases of the musculoskeletal system and connective tissue, and diseases of the respiratory system. Of the top ten herbal medicines prescribed by TCM physicians, the top nine herbal formulae and the top ten single herbs were associated with diseases of the respiratory system.

**Conclusion:**

This study identified patterns of TCM prescriptions for children and common disease categories treated with TCM. The results provide a useful reference for health policy makers and for those who consider the usage of TCM for children.

## Background

Traditional Chinese medicine (TCM) has generally been used not only in adults but also in pediatric patients by the Chinese population, as well as in Asia and many other countries around the world [1–4]. TCM may include acupuncture, traumatology, manipulative therapies, and moxibustion. Chinese herbal medicines are one of the most common used modes of TCM treatment [5, 6]. In Taiwan, TCM is an important part of health care and is reimbursed under the current National Health Insurance (NHI) system.

TCM application for pediatric disease is popular and widely used because most parents or caregivers believe that TCM, such as herbs, has a therapeutic effect without any harmful consequences [7]. Eighty percent of parents admitted to concurrent usage of TCM and conventional medicine for their children. In Singapore, herbal medicine was the most commonly used form of TCM, at 84.3% [8]. According to previous studies of two randomly sampled cohorts from the National Health Insurance Research Database in Taiwan, 22 and 22.5% of children used TCM in 2005 and 2010 respectively. Among them, herbal remedies were the most commonly used therapeutic approach, followed by manipulative therapy and acupuncture. In addition, there was an increasing trend of using herbal remedies (from 65.6 to 74.4%) and acupuncture (from 7.5 to 11.4%) from 2005 to 2010 [6]. In another national cohort of 112,159 children < 12 years, 18.3% had used TCM; school-age children (aged 6–12 years), preschool age children (3–5 years), and toddlers (1–2 years) were more likely to use TCM than infants [9]. However, children are vulnerable to drugs [10], so the dosage varies with age and sensitivity. Little is known about patterns of use of TCM in conditions related to childhood disease and adolescent disease; further study might increase the potential for traditional medicine to be used in Western countries. Accordingly, the computerized reimbursement database of the NHI, the National Health Insurance Research Database (NHIRD), stores longitudinal data on TCM; it also provides an optimal platform for the understanding of use patterns of TCM for children. Thus, the aim of our study is to analyze a random sample of this database and to determine TCM utilization patterns for children aged 18 years and under in Taiwan by analyzing NHI claims data from 2005 to 2013.

## Methods

### Data source

In the health care system in Taiwan, people are free to choose between Western medicine and TCM, and are allowed to visit either primary care clinics or hospitals without referral. Furthermore, NHI covers almost the entire Taiwanese population, accounting for 99.6% of the total population (23,737,000 beneficiaries at the end of 2015) [11, 12]. All TCMs are provided and prescribed by physicians, and all are covered by NHI. In addition, only licensed TCM physicians qualify for reimbursement. The insurance coverage for TCM in Taiwan includes Chinese herbal medicine (CHM), acupuncture, and traumatology manipulative therapy. TCM medical records files include medical care facilities and specialties; drugs and other management for treatment; and patients’ gender, date of birth, date of health care encounter, and unique identification number, which is used to protect the confidentiality of the patient’s individually identifiable information, as is the case for Western medication [13]. According to the construct of the database, the dataset reflected primary treatment only, meaning that if children were treated with both acupuncture and herbs, only the herbal treatment was recorded. Furthermore, three major diagnoses were coded in the International Classification of Disease, 9th Revision, and Clinical Modification (ICD-9-CM) format. These databases have previously been used for epidemiologic research and information on prescription use, diagnoses, and hospitalizations. In this study, a cohort of one million patients who were beneficiaries of the NHI program from January 1, 2005, to December 31, 2013, was randomly sampled. From the sampled group, we extracted children aged 18 years and younger for whom TCM was utilized. Patients with missing data on sex or birthdate were excluded.

Following strict confidentiality guidelines in accordance with personal electronic data protection regulations, the National Health Research Institute of Taiwan maintains an anonymous database of NHI reimbursement data that is suitable for research. In addition, this study was approved by the Ethics Review Board of Chang Gung Memorial Hospital, Chiayi Branch, Taiwan.

### Study subjects

For this study, we screened 178,617 eligible children aged from newborn to 18 years from the random cohort sample. The age was calculated by subtracting the birth date of the subject from December 31, 2013. We assembled a database of all outpatient department TCM records for 2005–2013 and included for final analysis the 112,889 subjects who had visited a TCM outpatient department at least once.

### Products of herbal formula (HF) and single herbs (SH)

TCM has developed over the past millennia and is a well-established component of the national health system in Taiwan. Its practice includes Chinese herbal remedies (CHR), acupuncture, and traumatology manipulative therapies; these are reimbursed by the NHI of Taiwan [13]. We downloaded the list of reimbursed Chinese herbal products from the website of the Bureau of NHI. Corresponding drug information on a specific mixture or name was then obtained from the Committee on Chinese Medicine and Pharmacy (CCMP) website, including the proportions of each constituent, date and period of drug approval, drug names, and manufacturers’ codes. Products of SH or HF are assigned with different drug registration numbers if produced by different manufacturers even though the constituents are the same. There are 2485 drug registration numbers for SH and 6639 drug registration numbers for HF, which involve 391 kinds of herbs based on 309 HF according to the unified formula announced by CCMP; all unified formulae were chosen from seven Chinese medicine books.

### Statistical analysis

We used SAS version 9.1 software (SAS Institute Inc., Cary, NC) for data analysis and descriptive statistics of drug utilization patterns. We linked drug registration numbers from the CCMP website to outpatient visit records of the study cohort. Then we analyzed the frequencies and percentages of the most frequently used HF and SH prescriptions. We calculated average daily doses and durations for each prescription. The TCM records include data indicating which SH or HF was prescribed, the prescription duration in days, and the dosage in grams. We used the following formula to calculate the average daily dose for SH and HF in the present study: (total dosage for SH or HF)/(total amount of prescription day for SH or HF) = average daily dose for SH or HF.

By using the ICD-9-CM of the first major diagnosis, records of visiting outpatient departments can be divided into different disease categories. We also analyzed the three most commonly used HF and SH for the five most common disease categories for the subjects.

## Results

A total of 112,889 out of 178,617 (63.2%) subjects that had TCM treatments between 2005 and 2013 were analyzed. Table 1 shows there were 11,448 children (10.1%) in the 0–5-year-old age group, 43,940 children (38.9%) in the 6–11-year-old age group, and 57,501 children (50.9%) in the 12–18-year-old age group who were treated as TCM outpatients. Analyses identified a significant difference in gender and age groups (*p <* 0.0001).

**Table 1:** TCM used among children in different age groups (*n* = 112,889)

Age (years)	0–5(preschool and under)		6–11(school)		12–18(adolescent)		Total		*p* value
	n	%	n	%	n	%	n	%	< 0.0001
Boys	6223	54.4^a^ (10.8)^b^	23,040	52.4 (40.0)	28,384	49.4 (49.2)	57,647	51.1 (100.0)	
Girls	5225	45.6 (9.5)	20,900	47.6 (37.8)	29,117	50.6 (52.7)	55,242	48.9 (100.0)	
Total	11,448	100.0 (10.1)	43,940	100.0 (38.9)	57,501	100.0 (50.9)	112,889	100.0 (100.0)	

Results are n and % using *x*^2^ test

^a^Column percentage

^b^Row percentage

Table 2 shows frequency distributions of TCM visits classified by major disease categories (according to ICD-9-CM codes). Of the 1,588,900 TCM outpatient visits among these children, 1,440,316 (90.6%) were treated with prescriptions of CHR, and 148,584 (9.4%) were treated with prescribed acupuncture and manipulative therapies. The top five categories of disease treated with TCM were: diseases of the respiratory system (42.9%); symptoms, signs, and ill-defined conditions (19.0%); diseases of the digestive system (10.6%); injury and poisoning (9.2%); and diseases of the skin and subcutaneous tissue (6.7%). These diseases or conditions accounted for more than 88.4% of all TCM visits. The top five major disease categories for which CHR was prescribed were diseases of the respiratory system (47.2%); symptoms, signs, and ill-defined conditions (21.0%); diseases of the digestive system (11.6%); diseases of the skin and subcutaneous tissue (7.4%); and disease of the genitourinary system (6.5%). These conditions accounted for more than 93.7% of CHR among all TCM visits. Moreover, injury and poisoning and diseases of the musculoskeletal system and connective tissue accounted for more than 97.4% of TCM visits using acupuncture and traumatology.

**Table 2:** Frequency distribution of TCM visits by major disease categories among children 18 years and younger

Major disease category	ICD-9-CM	Chinese herbal remedies		Acupuncture traumatology		Total of TCM	
		n (%)	Rank	n (%)	Rank	n (%)	Rank
Diseases of the Respiratory System	460–519	679,509(47.2)	1	1354(0.9)	3	680,863(42.9)	1
Symptoms, Signs, and Ill-Defined Conditions	780–799	301,712(21.0)	2	392(0.3)	7	302,104 (19.0)	2
Diseases of the Digestive System	520–579	167,701 (11.6)	3	245(0.2)	8	167,946 (10.6)	3
Diseases of the Skin and Subcutaneous Tissue	680–709	106,547 (7.4)	4	176(0.1)	10	106,723 (6.7)	5
Diseases of the Genitourinary System	580–629	94,168 (6.5)	5	2294(0.2)	4	96,462 (6.1)	6
Injury and Poisoning	800–999	25,573 (1.8)	6	121,101 (82.7)	1	146,674 (9.2)	4
Diseases of the Musculoskeletal System and Connective Tissue	710–739	24,109 (1.7)	7	21,523 (14.7)	2	45,632 (2.9)	7
Diseases of the Nervous System and Sense Organs	320–389	15,066 (1.1)	8	611(0.4)	5	15,677 (0.9)	8
Endocrine, Nutritional and Metabolic Diseases, and Immunity Disorders	240–279	7230 (0.5)	10	72(0.0)	11	7302(0.5)	10
Mental Disorders	290–319	6373 (0.4)	11	227(0.2)	9	6600(0.4)	11
Others^a^		12,328(0.9)	9	589(0.4)	6	12,917(0.8)	9
Total		1440,316(100)		148,584(100)		1,588,900 (100)	

^a^Others include ICD-9-CM code ranges 280–289, 630–677, 740–759, 760–779, V01-V82, E800-E999

Since CHR were the most common prescription, Table 3 displays the 10 most commonly prescribed HF and SH for children. It also includes the frequency of prescriptions, average daily doses (in grams) by age group, and average prescription duration (in days). In HF, the average daily dose was 2.5–3.1 g for children 0–5 years of age, 3.3–3.9 g for children 6–11 years of age, and 3.8–4.7 g for children 12–18 years of age; the average prescription durations were 5.5–7.2 days. The most commonly prescribed HF for children was Shin-Yi-Ching-Fey-Tang (SYCFT) (14.8%). The next four top HF were Xiao-Qing-Long-Tang (XQLT) (10.9%), Cang-Er-San (9.9%), Xin-Yi-San (XYS) (9.8%), and Ma-Xin-Gan-Shi-Tang (MXGST) (9.7%).

**Table 3:** Top ten herbal medicine prescribed by traditional Chinese physicians for children

Rank	Herbal formulae	Frequency (%)	Average daily doses (g), by age group			Average duration (days)	Single herb	Frequency (%)	Average daily doses (g), by age group			Average duration (days)
			0–5	6–11	12–18				0–5	6–11	12–18	
1	Shin-Yi-Ching-Fey-Tang	213,325 (14.8)	2.9	3.6	4.1	6.5	Gan-Cao (Radix Glycyrrhizae)	165,315 (11.5)	0.8	0.8	1.0	6.3
2	Xiao-Qing-Long-Tang	157,380 (10.9)	3.1	3.7	4.4	6.9	Jie-Geng (Radix Platycodi)	134,474 (9.3)	1.2	1.3	1.3	5.9
3	Cang-Er-San	142,826 (9.9)	2.8	3.3	3.8	6.4	Chuan-Bei-Mu (Bulbus Fritillariae Cirrhosae)	122,641 (8.5)	1.0	1.1	1.2	6.1
4	Xin-Yi-San	141,049 (9.8)	3.0	3.8	4.4	6.8	Yu-Xing-Cao (Herba Houttuyniae)	117,641 (8.2)	1.2	1.3	1.4	6.0
5	Ma-Xing-Gan-Shi-Tang	139,927 (9.7)	3.0	3.6	4.1	5.7	Xing-Ren (Semen Armeniacae)	102,099 (7.1)	1.2	1.2	1.3	6.0
6	Yin-Qiao-San	110,639 (7.7)	2.9	3.7	4.3	5.5	Huang-Qin (Radix Scutellariae)	100,846 (7.0)	1.3	1.4	1.5	6.2
7	Xin-Su-San	96,869 (6.7)	3.1	3.7	4.4	5.7	Chan-Tui (Cryptotympana atrata Fabr)	100,428 (7.0)	1.0	1.1	1.2	6.7
8	Ge-Gen-Tang	95,719 (6.6)	3.0	3.9	4.7	6.5	Bai-Zhi (Radix Angelicae Dahuricae)	94,423 (6.6)	1.2	1.3	1.4	6.6
9	Zhi-Sou-San	74,483 (5.2)	2.8	3.4	4.0	5.7	Cang-Er-Zi (Fructus Xanthii)	84,896 (5.9)	1.1	1.2	1.3	6.5
10	Jia-Wei-Xiao-Yao-San	70,066 (4.9)	2.5	3.8	4.4	7.2	Xin-Yi (Flos Magnoliae)	68,746 (4.8)	1.2	1.3	1.4	6.3

In SH, the average daily dose was 0.8–1.3 g for children 0–5 years of age, 0.8–1.4 g for children 6–11 years of age, and 1.0–1.5 g for children 12–18 years of age; the average prescription durations were 5.9–6.7 days. Gan-Cao (Radix Glycyrrhizae) (11.5%) was the most commonly prescribed SH for children. The next four most frequently prescribed SH were Jie-Geng (Radix Platycodi) (9.3%), Chuan-Bei-Mu (Bulbus Fritillariae Cirrhosae) (8.5%), Yu-Xing-Cao (Herba Houttuyniae) (8.2%), and Xing-Ren (Semen Armeniacae) (7.1%).

Regarding TCM used with CHR, Table 4 presents the most frequently used HF and SH for the five most common disease categories. SYCFT (25.7%), XQLT (18.8%), and XYS (18.1%) were the most common HF prescribed, and Gan-Cao (14.3%), Yu-Xing-Cao (13.3%), and Jie-Geng (13.1%) were the most common SH prescribed for respiratory system diseases. MXGST (14.1%), SYCFT (12.3%), and Xin-Su-San (11.5%) were the most frequently prescribed HF, and Jie-Geng (14%), Chuan-Bei-Mu (13.2%), and Gan-Cao (11.1%) were the most commonly prescribed SH for symptoms, signs, and ill-defined conditions. For diseases of the digestive system, Xiang-Sha-Liu-Jun-Zi-Tang (16.1%), Shen-Ling-Bai-Zhu-San (SLBZS) (16%), and Bao-He-Wan (15.2%) were the three most frequently used HF, and Shen-Qu (15.3%), Mai-Ya (14%), and Shan-Zha (11.2%) were the most commonly used SH. Xiao-Feng-San (XFS) (23.9%), Qing-Shang-Fang-Feng-Tang (QSFFT) (23.3%), and Zhen-Ren-Huo-Ming-Yin (17%) were the most frequently used HF, and Lian-Qiao (21.6%), Jin-Yin-Hua (16.7%), and Pu-Gong-Ying (15.4%) were the most frequently used SH for diseases of the skin and subcutaneous tissue. For genitourinary system diseases, Jia-Wei-Xiao-Yao-San (32.2%), Dang-Gui-Shao-Yao-San (18%), and Wen-Jing-Tang (16.7%) were the three most commonly used HF, while Xiang-Fu (21.7%), Yi-Mu-Cao (21.5%), and Yan-Hu-Suo (13.6%) were the most frequently prescribed SH.

**Table 4:** Three most common herbal medicine for five most common disease categories in children

Diseases categories	Herbal formulae		Single herb	
		Frequency (%)		Frequency (%)
Diseases of the Respiratory System	Shin-Yi-Ching-Fey-Tang	166,463 (25.7)	Gan-Cao (Radix Glycyrrhizae)	92,530 (14.3)
	Xiao-Qing-Long-Tang	121,716 (18.8)	Yu-Xing-Cao (Herba Houttuyniae)	86,083 (13.3)
	Xin-Yi-San	117,546 (18.1)	Jie-Geng (Radix Platycodi)	85,060 (13.1)
Symptoms, Signs, and Ill-Defined Conditions	Ma-Xing-Gan-Shi-Tang	39,987 (14.1)	Jie-Geng (Radix Platycodi)	39,658 (14.0)
	Shin-Yi-Ching-Fey-Tang	34,987 (12.3)	Chuan-Bei-Mu (Bulbus Fritillariae Cirrhosae)	37,366 (13.2)
	Xin-Su-San	32,792 (11.5)	Gan-Cao (Radix Glycyrrhizae)	31,433 (11.1)
Diseases of the Digestive System	Xiang-Sha-Liu-Jun-Zi-Tang	24,784 (16.1)	Shen-Qu (Massa Medicata Fermentata)	23,520 (15.3)
	Shen-Ling-Bai-Zhu-San	24,642 (16.0)	Mai-Ya (Fructus Hordei Germinatus)	21,490 (14.0)
	Bao-He-Wan	23,364 (15.2)	Shan-Zha (Fructus Crataegi)	17,203 (11.2)
Diseases of the Skin and Subcutaneous Tissue	Xiao-Feng-San	22,286 (23.9)	Lian-Qiao (Fructus Forsythiae)	20,107 (21.6)
	Qing-Shang-Fang-Feng-Tang	21,723 (23.3)	Jin-Yin-Hua (Flos Lonicerae)	15,588 (16.7)
	Zhen-Ren-Huo-Ming-Yin	15,831 (17.0)	Pu-Gong-Ying (*Taraxacum officinale*)	14,355 (15.4)
Diseases of the Genitourinary System	Jia-Wei-Xiao-Yao-San	25,164 (32.2)	Xiang-Fu (Rhizoma Cyperi)	16,977 (21.7)
	Dang-Gui-Shao-Yao-San	14,102 (18.0)	Yi-Mu-Cao (Herba Leonuri)	16,833 (21.5)
	Wen-Jing-Tang	13,049 (16.7)	Yan-Hu-Suo (Rhizoma Corydalis)	10,656 (13.6)

## Discussion

To date, this study is the most comprehensive investigation of TCM usage among children aged 18 years and younger. The advantages of the study are in the application of a random national-level sample to analyze and document comprehensive data gathered with unrestricted access, and is particularly valid for investigating TCM. However, children may be taken for treatment to other places, such as where folk medicine is practiced, and these are not included in the database. The number of TCM patients may be underestimated. That is the limitation of this study. The integration of TCM into the health care system in Taiwan has resulted in the NHIRD providing a large database of TCM usage with de-identified patient information. Previous studies have mainly consisted of questionnaire surveys or telephone interviews from hospitals or private clinics, mostly obtaining parents’ or caregivers’ information [7, 8, 14, 15]. Therefore, results were limited because of small sample sizes. Additionally, young children may be unable to express ideas clearly, and most of them are protected by parents or family caregivers without the chance to present their opinions freely. Therefore, these previous studies might only offer a limited picture of children’s TCM usage. Because TCM is reimbursed by Taiwan’s NHI, the results of this study could reveal a broad, less biased description and overview of children’s TCM usage.

We found adolescents occupied the greatest portion (50.9%) of all TCM age groups among the 112,889 child TCM patients selected. The findings of the most common TCM use group among children is consistent with previous studies [1, 6], which showed that as a child grows older, preference for pediatric TCM use increases. However, the prevalence of TCM use was represented differently; Huang et al. [6] showed 38.4% adolescent TCM use in 2005, and 42.7% in 2010. Our study showed 50.9% adolescent TCM use in 2005–2013. Several scholars identified the role of puberty in TCM treatment of adolescents, citing the physiological and behavioral changes associated with the attainment of reproductive competence as well as nonreproductive traits, such as social, emotional, and cognitive developmental factors associated with the transition from childhood to adulthood [16, 17]. However, another investigation [9] indicated that children’s age and parental TCM use were more strongly associated with TCM use, with parental TCM use being the most important factor influencing pediatric TCM use.

In our study, respiratory system diseases were the most common reason for children to visit TCM clinics, followed by symptoms, signs, and ill-defined conditions; digestive system diseases; injury and poisoning; and diseases of the skin and subcutaneous tissue. A previous study has revealed similar findings. Huang et al. [6] analyzed the use of TCM in children based on two random cohorts in 2005 and 2010 of NHIRD. The frequency distributions of diseases treated with TCM concurred with our study, but their study showed a different ordering of the reasons for children’s TCM visits. It indicated that respiratory system diseases remained the most common reason for children to visit TCM clinics in Taiwan in 2005 and 2010, followed by symptoms, signs, and ill-defined conditions; injury and poisoning; digestive system diseases; and diseases of the skin and subcutaneous tissue. Our findings differed from the previous study [9], indicating that musculoskeletal problems were the most common underlying medical conditions among children TCM users, followed by gastrointestinal problems and respiratory problems. Our results differed slightly when compared to the entire population of TCM users in Taiwan. As Chen’s study [14] showed, the top five most common reasons for TCM visits were diseases of the respiratory system; musculoskeletal system and connective tissue; symptoms, signs, and ill-defined conditions; injury and poisoning; and diseases of the digestive system. These findings could be explained by the frequency distributions of diseases for which TCM is commonly used. TCM contributes to the treatment of these diseases and plays a significant role in improving children’s health.

Additionally, a high-quality, pediatric asthma outpatient TCM clinic project was administered by Taiwan’s bureau of National Health Insurance and the National Union of Chinese Medical Doctors’ Association, ROC. This project benefited children with asthma [18, 19]. The project was piloted in 2006 for asthmatic children under 15 years old in and was fully implemented in 2013 for children 12 years old and under [20]. As a result of this program’s positive impacts for asthmatic children, the prevalence of TCM used for children may increase. Further studies are suggested to explore these potential effects.

Chinese herbal remedies (90.6%) (Table 2) comprised most TCM visits. Among them, respiratory system diseases were the leading ailment to be treated in our study. Furthermore, we found that most top ten herbal medicines including HF and SH prescribed by physicians (Table 3) were associated with treating respiratory disease. Thus, children and adolescents with respiratory disease were more likely to use TCM, especially CHR. Injury and poisoning were the most frequent conditions associated with acupuncture traumatology use, although acupuncture therapy occurred in few TCM visits (9.4%) in our study. In line with Lu, Chang, Sung, and Chen’s study [21], acupuncture is one of the most common treatment modalities used in injury management, and is likely to be common in patients with dislocations, sprains, and strains because of the effectiveness of TCM modalities on pain management and function improvement. Previous studies [22, 23] have reported that acupuncture helps in alleviating pain, chronic pain, and other conditions. Although the potential for acupuncture not to be accepted among pediatric populations exists because children are often afraid of needles, some studies have depicted pediatric acupuncture as both acceptable and feasible [24, 25]. However, different characteristics of disease may indicate different patterns of acupuncture use among children. Further studies are recommended to determine these patterns.

We further determined that SYCFT, XQLT, Xin-Yi-San in HF, and Gan-Cao, Yu-Xing-Cao, and Jie-Geng in SH, were the top three herbal medicines prescribed for diseases of the respiratory system (Table 4). Gan-Cao is commonly prescribed by TCM physicians for children because of the adjustment of taste [26, 27]. Symptoms, signs, and ill-defined conditions were the second most frequent diagnoses for TCM visits with prescriptions for CHR in our study. Furthermore, we found that HF and SH often used for diseases of the respiratory system were commonly used for symptoms, signs, and ill-defined conditions. Thus, we inferred that most patients who were treated for symptoms, signs, and ill-defined conditions suffered from diseases related to the respiratory system.

Traditionally, TCM physicians always chose therapeutic principles and methods based on syndrome differentiation theory and did not make specific diagnoses based on holistic considerations in patients with many different symptoms. Syndrome differentiation is a unique method for the diagnosis of disease in TCM, using the concepts of balance and harmony to analyze the patterns within the human body and make a diagnosis [28]. However, because younger children are unable to express themselves clearly, the diagnostic process is combined with clinical treatment into a holistic approach to determine patterns of dysfunction and treatment [29]. Moreover, there is no standard methodology in the disease coding system for TCM [13]. This may be why TCM physicians use the ICD-9-CM code for symptoms, signs, and ill-defined conditions instead of using specific diagnostic codes. It is important to develop more reliable coding systems for TCM diagnostic classifications.

Jia-Wei-Xiao-Yao-San (JWXYS) was the only exception in the top ten list in that it was not related to respiratory disease. It occupied tenth position (Table 3). However, it was the top commonly prescribed HF, followed by Dang-Gui-Shao-Yao-San (DGSYS) and Wen-Jing-Tang (WJT) for treating diseases of the genitourinary system (Table 4) in our study. Our finding is similar to previous studies [30, 31] that found DGSYS was the most commonly used HF, followed by JWXYS and WJT, to treat primary dysmenorrhea for women 13–25 years old, and 20–50 years old respectively. Dysmenorrhea is a common gynecological complaint of adolescent girls who often suffer from some level of discomfort due to menstruation [31, 32]. Our results correspond to a previous study [33], which concluded JWXYS was most often used for primary dysmenorrhea, followed by DGSYS and WJT, among HF; additionally, Xiang-Fu (*Cyperus rotundus* L.), Yi-Mu-Cao (Leonurus heterophyllus Sweet), and Yan-Hu-Suo (Corydalis yanhusuo W. T. Wang) were the most commonly used SH to treat diseases of the genitourinary system in our study. Furthermore, SH is often used as an adjuvant to HF; however, both SH of Xiang-Fu and Yi-Mu-Cao were prescribed more frequently than the HF of DGSYS in our study. According to TCM theory, the organs work together by regulating and preserving Qi (energy) and blood through the so-called channels and collaterals. Qi stagnation usually suggests that energy and information cannot move smoothly to or from its appropriate location [29]. Xiang-Fu is used to treat qi stagnation, and Yi-Mu-Cao is used to treat blood stasis [34]. It has been used to treat dysmenorrhea and irregular menstruation [30, 35].

XSLJZT is the most commonly prescribed HF for diseases of the digestive system. It is a common Chinese herbal prescription and used for the treatment of gastrointestinal diseases in Asian countries [36]. SLBZS enhances digestive function and removes moisture to clear digestive discomforts like diarrhea and distension [37, 38]. Shen-Qu was the most commonly prescribed SH to treat diseases of the digestive system in our study. In traditional Chinese medicine, Shen-Qu is used to treat conditions such as diarrhea, abdominal distension, and lack of appetite. Shen-Qu can also inhibit the activity of 3-hydroxy-3-methylglutaryl-coenzyme A (HMG-CoA) and maintain the balance of cholesterol in the body [39].

XFS was the most commonly prescribed HF, followed by QSFFT and Zhen-Ren-Huo-Ming-Yin, for diseases of the skin and subcutaneous tissue in our study. Previous studies [40, 41] found XFS was the most commonly prescribed Chinese herbal formula for atopic dermatitis and urticaria since it has an antipruritic effect for severe, refractory, extensive, and non-exudative atopic dermatitis [42]. Chien et al. [41] found that XFS was by far the most commonly prescribed Chinese HF for subjects with urticarial by analyzing the population-based CHM database from Taiwan. Furthermore, this herbal remedy helps address weepy, itchy, red skin lesions such as eczema, urticarial, psoriasis, and diaper rash. A previous study [43] found QSFFT was the most commonly used HF, followed by Zhen-Ren-Huo-Ming-Yin, among 279,823 CHM prescriptions to treat acne. We found Lian-Qiao, Jin-Yin-Hua, and Pu-Gong-Ying, often used as antibiotics in Chinese medicine [44, 45], were the most commonly prescribed SH for the treatment of diseases of the skin and subcutaneous tissue.

Chinese herbal products were invented about 40 years ago and have been utilized ever since [13]. There are no guidelines for children’s Chinese herbal product dosages. Thus, TCM doctors typically adjust the dosage according to their clinical experience, the patient’s age, and/or the patient’s body weight. Our research provides the average daily dose and treatment duration for the top ten herbal medicines commonly prescribed by TCM physicians for children in Taiwan.

## Conclusion

We conducted a nationwide, population-based study on the use of TCM in children 18 years of age and younger based on one randomly selected cohort from the 2005–2013 NHIRD healthcare claims data in Taiwan. TCM usage is common, with approximately 63.2% of children having been treated with it. The utilization increased with age, peaking in the 12- to 18-year-old age group. Respiratory system diseases were the most common reason for TCM treatment, and Chinese herbal remedies were the most commonly used TCM modality. Shin-Yi-Ching-Fey-Tang and Gan-Cao (Radix Glycyrrhizae) were the most commonly used formula and single herb. This study provides information about the prescription patterns of TCM and disease categories treated by TCM, which should be useful for health policy makers and for those who consider the usage of TCM for children.

## Abbreviations

CCMP: Committee on Chinese Medicine and Pharmacy
CHM: Chinese herbal medicine
CHR: Chinese herbal remedies
HF: Herbal formula
ICD-9-CM: International classification of disease, 9th revision, clinical modification
JWXYS: Jia-Wei-Xiao-Yao-San
MXGST: Ma-Xin-Gan-Shi-Tang
NHI: National Health Insurance
NHIRD: National Health Insurance Research Database
SH: Single herb
SLBZS: Shen-Ling-Bai-Zhu-San
SYCFT: Shin-Yi-Ching-Fey-Tang
TCM: Traditional Chinese medicine
XFS: Xiao-Feng-San
XQLT: Xiao-Qing-Long-Tang
XSLJZT: Xiang-Sha-Liu-Jun-Zi-Tang
XYS: Xin-Yi-San
